# Development of Protective Immunity in New Zealand White Rabbits Challenged with Bacillus anthracis Spores and Treated with Antibiotics and Obiltoxaximab, a Monoclonal Antibody against Protective Antigen

**DOI:** 10.1128/AAC.01590-17

**Published:** 2018-01-25

**Authors:** Lisa N. Henning, Sarah Carpenter, Gregory V. Stark, Natalya V. Serbina

**Affiliations:** a Elusys Therapeutics, Inc., Pine Brook, New Jersey, USA; b Battelle, West Jefferson, Ohio, USA

**Keywords:** anthrax, Bacillus anthracis, antitoxin, protective antigen, monoclonal antibodies, obiltoxaximab, immune memory

## Abstract

The recommended management of inhalational anthrax, a high-priority bioterrorist threat, includes antibiotics and antitoxins. Obiltoxaximab, a chimeric monoclonal antibody against anthrax protective antigen (PA), is licensed under the U.S. Food and Drug Administration's (FDA's) Animal Rule for the treatment of inhalational anthrax. Because of spore latency, disease reemergence after treatment cessation is a concern, and there is a need to understand the development of endogenous protective immune responses following antitoxin-containing anthrax treatment regimens. Here, acquired protective immunity was examined in New Zealand White (NZW) rabbits challenged with a targeted lethal dose of Bacillus anthracis spores and treated with antibiotics, obiltoxaximab, or a combination of both. Survivors of the primary challenge were rechallenged 9 months later and monitored for survival. Survival rates after primary and rechallenge for controls and animals treated with obiltoxaximab, levofloxacin, or a combination of both were 0, 65, 100, and 95%, and 0, 100, 95, and 89%, respectively. All surviving immune animals had circulating antibodies to PA and serum toxin-neutralizing titers prior to rechallenge. Following rechallenge, systemic bacteremia and toxemia were not detected in most animals, and the levels of circulating anti-PA IgG titers increased starting at 5 days postrechallenge. We conclude that treatment with obiltoxaximab, alone or combined with antibiotics, significantly improves the survival of rabbits that received a lethal inhalation B. anthracis spore challenge dose and does not interfere with the development of immunity. Survivors of primary challenge are protected against reexposure, have rare incidents of systemic bacteremia and toxemia, and have evidence of an anamnestic response.

## INTRODUCTION

Inhalational anthrax is caused by spores of Bacillus anthracis, a Gram-positive bacterium found in soils all over the world. B. anthracis is a top priority biowarfare category A agent (1) and is considered a high-priority public threat (2). Several antibiotics, such as ciprofloxacin, levofloxacin, and doxycycline, are FDA approved for the treatment of inhalational anthrax and must be given for 60 days to ensure complete spore clearance (3). Following the 2001 U.S. anthrax bioterrorism attack, 11 people developed inhalational anthrax, and 5 people died despite aggressive treatment with multiple antibiotics and supportive therapy (4). The high mortality rate among victims of inhalational anthrax brought forth the need to develop therapeutics against inhalational anthrax that could be adjunctive to antibiotics.

Virulence of B. anthracis depends critically on the secretion of lethal toxin (LT) and edema toxin (ET) composed of the enzyme moiety lethal factor (LF) and edema factor (EF), respectively, and the common binding component, protective antigen (PA). Toxins contribute to pathogenesis through mediating tissue cytotoxicity and suppressing host immune responses (5), and PA neutralization is effective in preventing the establishment and progression of inhalational anthrax in animal models (6). The level of anti-PA IgG at the time of challenge is the single most accurate correlate of protection against inhalation anthrax (7), and seroconversion has been extrapolated to predict survival probability in humans (8, 9). Currently, the CDC recommends that antitoxins against PA should be added to antimicrobial drug treatment for any patient for whom there is a high level of clinical suspicion for systemic anthrax (3). Obiltoxaximab (ETI-204) is a chimeric IgG1(κ) monoclonal antibody that binds with high affinity to PA and prevents its association with cellular receptors (10). The efficacy of obiltoxaximab has been demonstrated in animal models (11, 12), and it was recently licensed under the FDA's Animal Rule for the treatment of inhalational anthrax due to B. anthracis in combination with appropriate antibacterial drugs and for prophylaxis of inhalational anthrax when alternative therapies are not available or are not appropriate (13).

The administration of antibiotics can lead to spore latency (14), and reemergence of infection after discontinuation of treatment or due to noncompliance is a significant concern with inhalational anthrax infection. Thus, it is desirable to know whether adaptive immunity develops under differing treatment regimens and whether this memory immunity is protective against reexposure. Here, we examined the immune status of New Zealand White (NZW) rabbits challenged with a lethal dose of B. anthracis spores and treated with antibiotics, obiltoxaximab, or a combination of both. The goal of the study was to evaluate the development of adaptive immune responses in spore-challenged animals given mono- or combination therapy and to compare protective immune statuses following differing treatment regimens. To our knowledge, this is the most comprehensive evaluation of adaptive immunity in anthrax-infected and treated animals conducted to date.

## RESULTS

### Establishment of memory immunity in NZW rabbits.

The overview of the study design is shown in Table 1. In phase 1, NZW rabbits were spore challenged and treated 30 h later with either a placebo, a single 16 mg/kg dose of obiltoxaximab, the first of 3 daily doses of levofloxacin, or obiltoxaximab in combination with levofloxacin. Published data from patients with cutaneous anthrax (15) suggest that seroconversion to detectable anti-PA IgG responses occurs only in the patients with evidence of systemic infection. In B. anthracis spore-challenged NZW rabbits, the mean time to first appearance to PA in blood was approximately 30 h (16). Thus, 30 h was selected as the treatment time to ensure that the majority of treated animals had evidence of systemic disease. The pretreatment disease parameters are summarized in Table 2. The mean challenge spore doses were comparable in all groups. Systemic infection, as measured by presence of blood bacteremia, was detected in 85% to 90% of the rabbits receiving active treatment, and the geometric means for bacteremia were similar among groups that received levofloxacin, obiltoxaximab, or a combination of both. For all four groups, PA levels were below the level of quantitation in most animals.

**TABLE 1:** Study design

Group	*n* or survivor group	Targeted LD_50_ challenge dose	Obiltoxaximab (mg/kg, i.v.)^*a*^	Levofloxacin (mg/kg/day, p.o.) × 3 days^*b*^	Treatment time PMC (h)^*c*^
Phase 1 (primary challenge)					
1	20	200	16	0 (vehicle)	30
2	20	200	0 (saline)	50	30
3	20	200	16	50	30
4	8	200	0 (saline)	0 (vehicle)	30
Phase 2 (rechallenge)					
1	Survivor group 1 (phase 1)	200	None	None	NA
2	Survivor group 2 (phase 1)	200	None	None	NA
3	Survivor group 3 (phase 1)	200	None	None	NA
5	12	200	None	None	NA

a i.v., intravenous.

b p.o., orally.

c PMC, post-mean challenge; NA, not applicable.

**TABLE 2:** Pretreatment characteristics

Group	Dose and route	Spore challenge LD_50_ (mean [SD])	Bacterial burden PTT (CFU/ml)^*a*^		PA PTT (ng/ml)^*a*^	
			No. positive/total no. (%)	Geometric mean (95% CI)	No. positive/total no. (%)	Geometric mean (95% CI)
1	Obiltoxaximab (16 mg/kg i.v.) + water (0 mg/kg/day p.o. × 3 days)	238.1 (58.6)	17/20 (85)	5.9E+02 (1.24E+02, 2.8E+03)	4/19 (21)	6.8 (4.8, 9.6)
2	Saline (0 mg/kg i.v.) + levofloxacin (50 mg/kg/day p.o. × 3 days)	209.2 (41.0)	18/20 (90)	3.4E+02 (9.7E+01, 1.2E+03)	1/20 (5)	5.5 (4.3, 7.0)
3	Obiltoxaximab (16 mg/kg i.v.) + levofloxacin (50 mg/kg/day p.o. × 3 days)	207.1 (37.4)	17/20 (85)	5.9E+02 (1.1E+02, 3.1E+03)	4/19 (21)	6.5 (4.9, 8.7)
4	Saline (0 mg/kg i.v.) + water (0 mg/kg/day p.o. × 3 days)	221.9 (47.0)	4/8 (50)	2.4E+01 (1.9E+00, 3.1E+02)	0/8 (0)	NA

a PTT, prior to treatment; CI, confidence interval; NA, not applicable.

**FIG 1:**
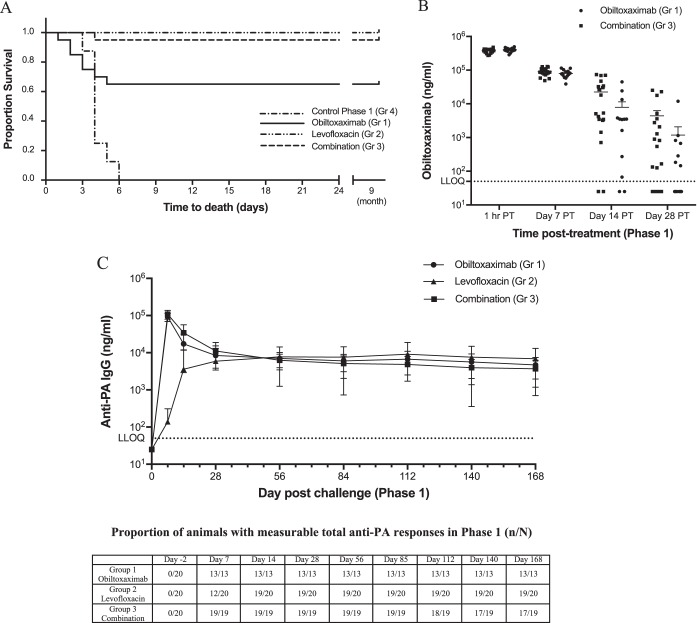
Kinetics of anti-PA antibodies in challenged and treated animals. NZW rabbits were aerosol challenged with targeted 200 LD_50_ of B. anthracis spores and treated with obiltoxaximab (circles), levofloxacin (triangles), or a combination of both (squares) at 30 h PC. (A) Kaplan-Meier curves representing time to death from challenge and survival data for each group are shown. (B and C) Serum samples were collected at the indicated times relative to treatment (posttreatment [PT]) or challenge for a specific assessment of obiltoxaximab (B) or nonspecific assessment of all circulating anti-PA IgGs (C). (A) Each symbol represents individual animal values. (B) Vertical lines indicate means and standard error for each group at each indicated time point. (C) Vertical lines indicate means and standard deviations for each group at each indicated time point. Numbers of animals with measurable total anti-PA IgG levels (n) and the total numbers of animals surviving to each time point (N) are shown on the bottom. Total anti-PA pool is composed of endogenous rabbit antibodies to PA as well as circulating obiltoxaximab. Dotted lines indicate lower limit of quantitation for each assay. For statistical computations, levels below limit of detection were replaced with ½ LLOQ. The LLOQ was 50 ng/ml in each assay.

All eight phase 1 control animals succumbed to B. anthracis infection, while 65% (13/20), 100% (20/20), and 95% (19/20) of the animals treated with obiltoxaximab, levofloxacin, and obiltoxaximab and levofloxacin, respectively, survived to rechallenge (Fig. 1A). The proportion of animals surviving the primary challenge in groups 1 through 3 was significantly greater than that of the phase I control group (Table 3). Complete resolution of bacteremia occurred by day 7 following primary challenge for all rabbits that survived to that time point and was maintained through the phase 1 observation period.

**TABLE 3:** Phase 1 survival rates

Group	Dose and route	Time of administration (h postchallenge)	Survival % (no. survived/no. treated)	*P* value^*a*^	95% CI^*b*^
1	Obiltoxaximab (16 mg/kg i.v.) + water (0 mg/kg/day p.o. × 3 days)	30 ± 4	65 (13/20)	0.0018	(0.156, 0.846)
2	Saline (0 mg/kg i.v.) + levofloxacin (50 mg/kg/day p.o. × 3 days)	30 ± 4	100 (20/20)	0.0010	(0.631, 1.000)
3	Obiltoxaximab (16 mg/kg i.v.) + levofloxacin (50 mg/kg/day p.o. × 3 days)	30 ± 4	95 (19/20)	0.0010	(0.604, 0.999)
4	Saline (0 mg/kg i.v.) + water (0 mg/kg/day p.o. × 3 days)	30 ± 4	0 (0/8)	NA	NA

a Boschloo test (with Berger-Boos modification of gamma = 0.001) compared to control. NA, not applicable.

b Exact 95% confidence interval of difference in survival rate.

### Development of anti-PA antibody responses following challenge and treatment.

Levels of circulating anti-PA IgG titers were measured using a protein A/G method that is species nonspecific and detects both endogenous rabbit antibodies to PA and obiltoxaximab. To understand the relative contributions of endogenously secreted anti-PA antibodies and obiltoxaximab to the overall anti-PA IgG profile, obiltoxaximab was additionally measured by a human IgG-specific assay that detects obiltoxaximab but not rabbit antibodies. In infected rabbits, intravenously administered obiltoxaximab has a serum half-life of approximately 1 day (17) and is cleared or significantly diminished in circulation by day 28 postchallenge (Fig. 1B, and data not shown). In phase 1 of the study, anti-PA antibody levels were not detected prior to challenge but were elevated by day 7 postchallenge in all groups (Fig. 1C). On days 7 and 14 following challenge, the mean anti-PA IgG levels in the group treated with levofloxacin only (group 2) were lower than those in the group treated with obiltoxaximab or the group treated with obiltoxaximab and levofloxacin (Fig. 1C). These differences were attributed to the detection of only endogenous anti-PA IgG in group 2 (levofloxacin monotherapy group). In contrast, both endogenous antibodies and obiltoxaximab were detected in group 1 and 3 animals during the first 4 weeks following challenge due to the nonspecific format of the assay. By 28 days following challenge, obiltoxaximab levels were either below detection or markedly reduced compared to previous time points (Fig. 1B). On day 56 postchallenge, all rabbits had circulating anti-PA IgGs, except for one animal in group 2 that did not have detectable anti-PA IgG at any phase 1 time point. Comparable levels of anti-PA IgG were observed in all groups, and those levels were maintained for the duration of the observation period, with only 2 rabbits in group 3 having no detectable anti-PA IgGs at days 112, 140, and 168 (Fig. 1C).

**FIG 2:**
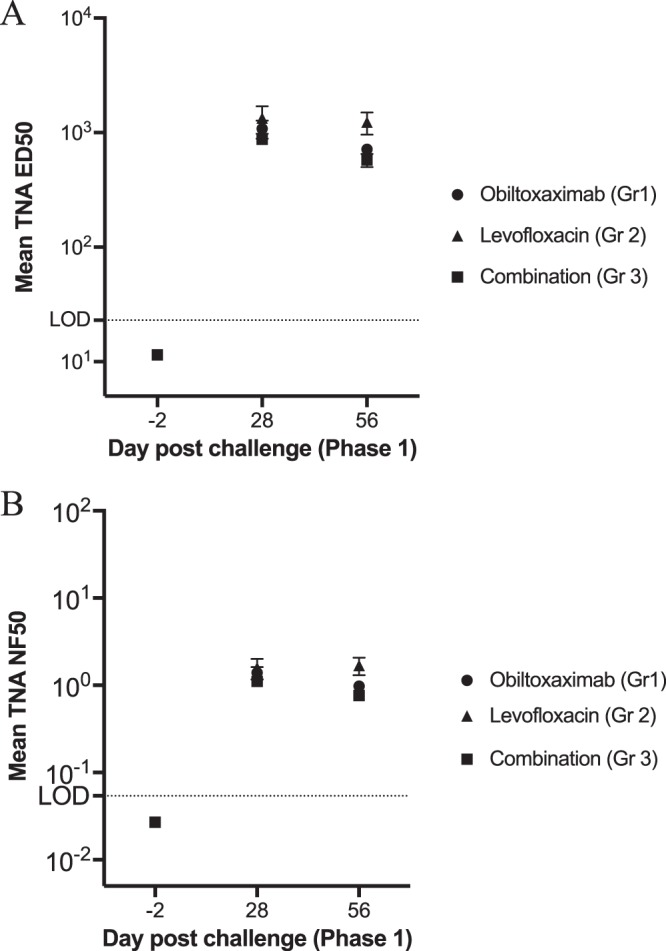
Toxin-neutralizing activity in serum of challenged and treated animals (phase 1). Serum samples were collected immediately prior to primary challenge or at the indicated times postchallenge for the assessment of TNA ED_50_ (A) and NF_50_ (B). Vertical lines indicate means and standard error. For statistical computations, TNA ED_50_ and NF_50_ levels below the limit of detection were replaced with 11.5 and 0.027 (½ of each respective LOD).

Toxin-neutralizing antibody levels were measured via the TNA in the serum of surviving rabbits on days 28 and 56 postchallenge in phase 1. The primary TNA endpoints were the 50% effective dilution (ED_50_) and the 50% neutralization factor (NF_50_). As expected, the TNA ED_50_ (Fig. 2A) and NF_50_ (Fig. 2B) were below the limits of detection for all animals in groups 1 through 3 prior to primary challenge. By day 28 following primary challenge, all surviving animals had developed a TNA ED_50_ and NF_50_ titer which continued to be detected at 2 months following challenge (Fig. 2), except for one animal in group 2 that also did not have a measurable anti-PA IgG response in phase 1 (discussed above). However, this rabbit had a quantifiable anti-PA IgG titer immediately prior to rechallenge, albeit in the absence of detectable TNA activity.

### Survival following rechallenge.

Prior to rechallenge, the mean body weights, ages, anti-PA IgG levels, and TNA ED_50_ and NF_50_ titers were comparable across all phase 1 treatment groups (Table 4). All primary challenge survivors (groups 1 through 3) had measurable anti-PA IgG levels and TNA activity prior to the rechallenge (day −7 or 9 months post-primary challenge), with the exception of two rabbits in group 3 and one rabbit in group 2 (discussed earlier). In two group 3 rabbits, anti-PA IgG levels were detectable until 4 and 5 months (days 140 and 168) post-primary challenge and declined thereafter.

**TABLE 4:** Phase 2 demographics

Group	*n*	Age (mean [SD]) (yr)	Wt (mean [SD]) (kg)^*a*^	Geometric mean anti-PA IgG (ng/ml [%CV])^*b*^	Geometric mean TNA ED_50_/NF_50_^*b*^	Spore challenge LD_50_ (mean [SD])
1 (survivor group 1, phase 1)	13	17.0 (0.00)	3.8 (0.43)	2,700 (77)	256/0.321	238.1 (58.6)
2 (survivor group 2, phase 1)	20	17.0 (0.00)	3.9 (0.41)	3,140 (212)	383/0.558	209.2 (41.0)
3 (survivor group 3, phase 1)	19	17.0 (0.00)	3.9 (0.39)	1,520 (343)	163/0.287	207.1 (37.4)
5 (naive controls)	12	11.3 (0.98)	3.9 (0.12)	<LOQ (0)	NA	221.9 (47.0)

a Measured on day of challenge.

b Measured only in phase 1 survivors. LOQ, limit of quantitation = 50.0 ng/ml for anti-PA IgG ELISA; NA, not applicable.

**FIG 3:**
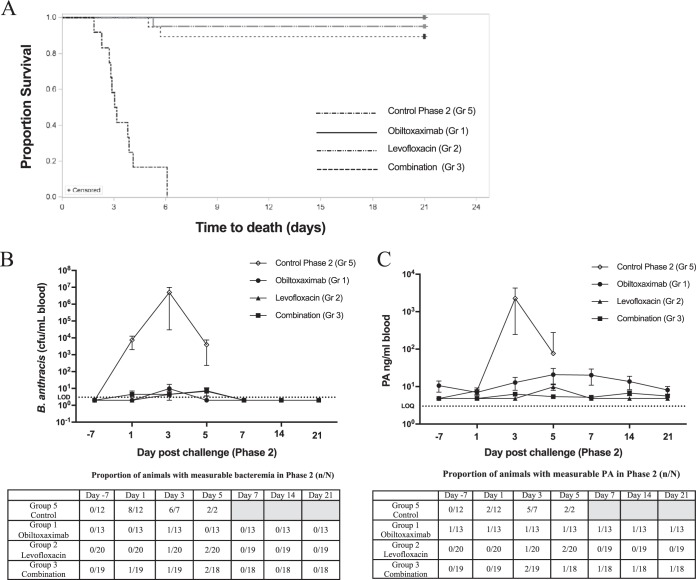
Survival and systemic disease development in immune animals following rechallenge. Nine months after primary challenge, phase 1 survivors and 12 additional naive controls were challenged with targeted 200 LD_50_ of B anthracis spores and monitored for 21 days. (A) Kaplan-Meier curves representing time to death from challenge and survival data for each group are shown. (B and C) Whole-blood samples were collected prior to treatment (day [D] −7) or at indicated times postrechallenge (PC) for the assessment of quantitative bacteremia (B) and circulating free PA (C). Shown are the means and standard error of the mean (SEM) for bacteremia and free PA at each indicated time point. Dotted lines represent limit of detection (LOD) for bacteremia or limit of quantitation (LOQ) for PA. Numbers of animals with measurable levels for each parameter (n) and the total numbers of animals surviving to each time point (N) are indicated at the bottom. Shaded areas indicate that no animals survived to that time point. For statistical computations, bacteremia levels below the LOD were replaced with 2 CFU/ml (½ LOD), and PA levels below limit of quantitation (LOQ) were replaced with 4.84 ng/ml (½ LOQ).

All phase 1 survivors and 12 naive approximately age- and weight-matched control rabbits received rechallenge with a targeted dose of 200 LD_50_ of B. anthracis spores. The administered spore doses were comparable across all groups, and no treatment was administered during phase 2. All 12 phase 2 naive control animals succumbed to B. anthracis infection, while 100% (13/13), 95% (19/20), and 89% (17/19) of the animals treated with obiltoxaximab, levofloxacin, and obiltoxaximab and levofloxacin during primary challenge, respectively, survived to the end of the phase 2 observation period (Fig. 3A). The proportion of surviving animals in each phase 1 obiltoxaximab treatment group was significantly greater than that in the phase 2 control group at the 0.025 level (*P* < 0.0001 for each group compared to the control). There were no significant differences in the proportion of animals that survived rechallenge among groups 1, 2, and 3. The times from rechallenge to death were also not significantly different among the groups.

### Development of systemic disease following rechallenge.

Bacteremia and toxemia development following rechallenge were examined. In the control group, 75% (8/12) of the rabbits were bacteremic 24 h following challenge, and bacteremia levels rose exponentially in all rabbits (Fig. 3B), with mean terminal bacteremia levels of 7.9 ×10^6^ CFU/ml. All control animals were bacteremic at least at one time point following challenge. In contrast, B. anthracis was not detected in blood cultures at nearly all time points for animals in groups 1 through 3 that had been challenged 9 months prior to a second challenge. In addition, positive B. anthracis cultures in survivors were transient and only present in low numbers (1/13 in group 1, 1/19 in group 2, and 2/17 in group 3) (Fig. 3B). The animals in groups 2 and 3 that succumbed to disease during the rechallenge all had positive blood terminal cultures (1.7 × 10^2^ CFU/ml, group 2 nonsurvivor; 4.5 × 10^2^ CFU/ml and 3.1 × 10^6^ CFU/ml, group 3 nonsurvivors).

In addition, bacterial dissemination to the peripheral tissues (bronchial lymph node, brain, liver, and spleen) was examined in all survivors at the end of the study and in all phase 2 nonsurvivors. B. anthracis was detected in at least three of the tissues evaluated in all animals that succumbed during phase 2. In contrast, no animals that survived rechallenge had a positive culture from any tissue assessed.

The presence of circulating PA was examined in each group prior to and following phase 2 challenge (Fig. 3C). The phase 2 naive control animals had detectable PA levels as early as 24 h after challenge, and all control animals had elevated PA levels prior to succumbing to infection. In contrast, the majority of rechallenged animals did not have detectable PA throughout the phase 2 period (Fig. 3C). Prior to rechallenge (day −7), all animals in groups 1 through 3 were negative for PA, with the exception of one animal in group 1 (obiltoxaximab phase 1 treatment). The PA levels for this animal were above the limit of quantitation and at a constant low level until the scheduled sacrifice on day 21. Persistent PA detection in this animal may have been related to cross-reactivity with circulating anti-obiltoxaximab antibodies due to the assay format. B. anthracis was not observed in the blood cultures at any phase 2 time points for this animal (including day −7), and the levels of PA detected in this animal were low and did not change significantly throughout phase 2. In addition, a transient PA increase following rechallenge was observed in several group 2 and 3 animals. Two animals in group 2 were positive for PA on day 5 post-secondary challenge, and both animals were also positive for bacteremia at this time point. One of these animals survived to the scheduled sacrifice, while the second PA-positive animal succumbed shortly after day 5 time point. In addition, two group 3 animals had quantifiable PA levels at the day 3 time point, and both animals succumbed to infection.

### Boost in memory immunity after rechallenge.

**FIG 4:**
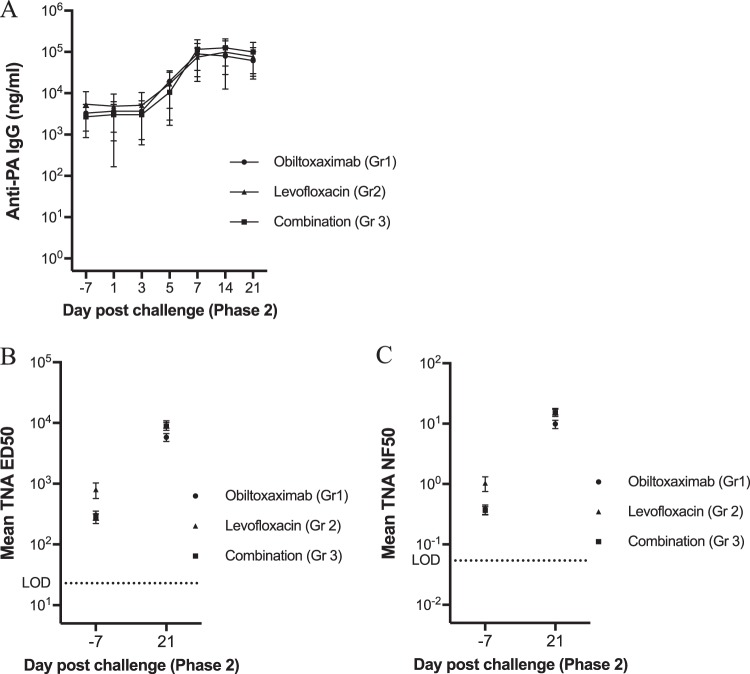
Assessment of anti-PA IgG and TNA responses following rechallenge. Whole-blood samples were collected prior to rechallenge (D −7) or at indicated times postrechallenge (PC) for the assessment of circulating anti-PA IgG levels (A), TNA ED_50_ (B), and TNA NF_50_ (C). Shown are means and standard deviation (SD) (A) and SEM (B and C) at each indicated time point. Dotted lines represent the LLOQ (anti-PA IgG) and LOD (TNA ED_50_ and NF_50_).

Figure 4 summarizes the results for anti-PA IgG and TNA levels by time point from day −7 through day 21 postrechallenge. There were no significant differences among the rechallenged groups at any of these time points. With the exception of two group 3 rabbits, all treated animals that survived the primary challenge had measurable anti-PA IgG levels prior to the rechallenge, and those levels remained unchanged for the first 3 days after challenge. The anti-PA IgG levels increased for all treatment groups by 5 days postrechallenge and remained elevated through the end of the in-life period.

On day 21 postrechallenge, the TNA ED_50_ and NF_50_ titers increased from the prechallenge baseline in all surviving animals (Fig. 4B and C and Table 5). To determine if the TNA levels were significantly different among the groups, an ANOVA model was fitted to the log_10_-transformed TNA ED_50_ by phase 2 time point. There were no significant differences among the groups for TNA ED_50_ and NF_50_ titers either before or after rechallenge (Table 5).

**TABLE 5:** Phase 2 ANOVA results for TNA by time point

TNA results by day postrechallenge^*a*^	Geometric mean			Group effect *P* value
	Group 1	Group 2	Group 3	
TNA ED_50_				
−7	256	383	163	0.1031
21	5,020	6,000	3,670	0.6469
TNA NF_50_				
−7	0.321	0.558	0.287	0.0938
21	8.36	14.1	12.7	0.0633

a Phase 2 rechallenge occurred 9 months following primary challenge.

## DISCUSSION

We examined the development of protective immunity in rabbits challenged with a lethal dose of B. anthracis spores and treated with obiltoxaximab administered intravenously alone or in combination with antibiotics. This study models a postexposure scenario of treatment with an antibiotic and/or anthrax antitoxin following inhalational exposure to spores and the development of systemic disease. Currently, several monoclonal and polyclonal antitoxins are stockpiled by the U.S. Government, and antitoxins are recommended for use in conjunction with antimicrobial therapy in patients suspected of having systemic anthrax infection (18).

Early during infection, anthrax toxins suppress host immunity through the impairment of innate immune cells and stifling efficient priming and effector functions of adaptive immunity (5, 19, 20). Therefore, it is plausible that toxin inhibition by obiltoxaximab administered alone or in combination with antibiotics may improve immune function following infection and promote the establishment of long-term immunological memory. Alternatively, it could be envisioned that PA-binding antitoxins may sequester free PA from antigen-presenting cells and interfere with priming of anti-PA responses. Thus, the objectives of this study were to establish that treatment with obiltoxaximab alone or in combination with antibiotics following primary anthrax challenge results in the development of protective immune and to compare the magnitude of recall immune responses in animals administered different treatment regimens.

To assess protective immunity following challenge and treatment, we examined survival and the development of bacteremia and toxemia after immune animals were reexposed to a lethal dose of spores. In addition, the kinetics of endogenously produced anti-PA IgGs were characterized throughout the study. The results of this study are consistent with the hypothesis that obiltoxaximab administration does not interfere with priming of adaptive immunity and that animals treated with obiltoxaximab after exposure to B. anthracis develop an immune response protective against rechallenge. In addition, we demonstrate that humoral immune responses (as measured by circulating rabbit anti-PA IgGs) develop comparably in animals given antibiotics, antitoxin, or a combination of both. Cumulatively, our study outcomes project that toxin neutralization via the administration of either an antitoxin that directly removes toxins from circulation or an antibiotic that limits toxin production by bacterial cells enables host survival and progression toward the development of adaptive immune responses. These results are in alignment with the published analyses of immune responses in human victims of anthrax outbreak following antibiotic treatment (21).

In phase 1 of the study, rabbits were treated with obiltoxaximab, levofloxacin, or a combination of obiltoxaximab and levofloxacin at 30 h following spore challenge. The treatment time was selected based on a previously published study where the mean time to PA appearance in blood was 30 h postchallenge (16). While the majority of animals in our study were negative for blood PA at the time of treatment administration, we attribute this discrepancy to the differences in the sensitivities of assays for PA measurement employed in our study and in the study by Migone et al. (16) (limits of detection of 9.68 ng/ml and 0.6 ng/ml, respectively). Notably, 85 to 90% of the animals in the treatment groups were positive for blood bacteremia in our study, and it can be projected that more animals would have had positive PA measurements if a more sensitive assay were employed. The observed phase 1 survival rates were consistent with previously published results (12). All treated animals had evidence of endogenous immune responses to PA, and there was no evidence of reemergence of disease due to spore latency in any of the treatment groups. Following rechallenge, survivors of primary challenge were highly protected against reexposure, irrespective of the treatment administered. Toxemia and bacteremia were detected in less than 10% of the rechallenge survivors and were transient and only present at low concentrations, suggesting that memory immunity was effective in curbing the development of systemic disease upon reexposure. In addition, none of the animals that survived rechallenge had evidence of tissue bacterial burden.

The development of anti-PA antibody responses and the functional ability of serum to neutralize lethal toxin were characterized in survivors of the primary challenge. Based on observations in survivors of the 2001 U.S. anthrax bioterrorism attack (21), it was anticipated that the anti-PA IgG levels would steadily decrease over the 6-month period following primary challenge. However, the anti-PA IgG titers for nearly all animals remained stable between 2 and 9 months post-primary challenge. Immediately prior to rechallenge, all but two rabbits had an anti-PA IgG titer, and levels of circulating anti-PA antibodies or toxin-neutralizing activity were comparable between rabbits in the obiltoxaximab treatment arms and levofloxacin-alone arm. Notably, within 5 days following rechallenge, anti-PA IgG levels for nearly all animals increased compared to the rechallenge baseline, suggesting that these animals had developed a memory B-cell response following primary challenge. The levels of TNA NF_50_ in all treatment groups were lower at rechallenge than the predicted level of 0.56, corresponding to a 70% probability of survival in rabbits vaccinated with Anthrax Vaccine Absorbed (22). It is plausible that antibodies elicited by active infection may be more effective than by a vaccine, or infection may activate other immune components, such as antibodies to non-PA antigens or cellular immunity.

While nearly all survivors of rechallenge had anti-PA IgG titers and the functional ability to neutralize toxins, two of the survivors had notable results. An anti-PA IgG titer declined in one combination-treated rabbit by 4 months post-primary challenge, but this animal had a quantifiable anti-PA IgG titer starting 7 days postrechallenge and did not have bacteremia at any point throughout the rechallenge period. In contrast, one levofloxacin-treated animal did not have detectable anti-PA IgG or TNA activity throughout the phase 1 period. This animal developed an anti-PA IgG titer approximately 9 months post-primary challenge (in the absence of quantifiable TNA) and continued to have a quantifiable anti-PA IgG titer throughout the rechallenge period. While this animal survived, there was a detectable bacteremia for this animal at days 3 and 5 postrechallenge.

The anti-PA IgG and TNA results for 2 nonsurvivors of the rechallenge were examined closely. One nonsurvivor was treated with obiltoxaximab and levofloxacin in phase 1 and had an anti-PA IgG titer that decreased after 2 months and was undetectable by 5 months postchallenge. The anti-PA IgG titer in this animal remained below the limit of quantitation until 3 days postrechallenge, when the animal succumbed to infection. In contrast, one rechallenge nonsurvivor from the levofloxacin treatment group had a detectable anti-PA IgG titer and TNA activity prior to rechallenge. This animal died despite having an anti-PA IgG titer through 5 days postrechallenge. These results suggest that the presence of circulating anti-PA IgG titers and toxin neutralization titers after priming of adaptive immunity may not be always sufficient to protect against repeat exposure to spores.

To our knowledge, this study provides the most comprehensive longitudinal investigation of anti-PA antibody responses in rabbits following B. anthracis infection and treatment. Previous study found that rabbits administered a human monoclonal antibody to PA at the time of spore challenge were protected against rechallenge 5 weeks later (23). However, definitive conclusions regarding the role of endogenous immunity could not be drawn from this study, because injected antibody was still found in circulation at the time of rechallenge. It should be noted that our study has some limitations. All challenges were conducted with a uniformly high spore dose, and no conclusions can be drawn regarding the relationship between spore dose and anti-PA IgG levels. In addition, only responses to PA were investigated, and anti-PA IgG and TNA were the sole correlates of protection. Following infection and treatment, adaptive responses against other bacterial and toxin epitopes may develop and contribute to protection (24–27). Analysis of cross-species survival data across multiple anthrax vaccination studies suggested that TNA is highly predictive of survival in animal models, and the cross-species results support that extrapolation from animal to human data may be informative (8, 9). Finally, the results of animal studies should not be overinterpreted in the absence of further confirmation through analyses of human data gathered during inhalational anthrax outbreaks. However, we believe that the results obtained in the rabbit model of inhalational anthrax reasonably predict what would occur in humans under similar circumstances. NZW rabbits have been a valuable model for the testing and development of vaccines and therapeutics against anthrax and are an accepted model for the approval of anthrax therapeutics under the FDA's Animal Rule (28). The course of inhalational anthrax in the rabbit model is reasonably comparable to that of human disease, and rabbits are expected to react with a response predictive for humans. Protective antigen is thought to have immunomodulatory activity toward human neutrophils and cells of mononuclear origin function and has been proposed to directly suppress human innate immune responses (19). The results of the studies with toxin-deficient strains of B. anthracis in NZW rabbits established that protective antigen plays a major role in the anthrax pathogenesis in rabbits through modulating innate host effector mechanisms with possible immunomodulating effect on heterophils, the rabbit equivalent of human neutrophils (29). While additional future studies can address the specific mechanisms of PA-induced immunosuppression in cells of human and rabbit origin, the interference of toxin with the innate immune system has been plausibly established in both species, and analyses of immune responses in treated rabbits have been warranted. Therefore, we believe that our findings have relevance to understanding the immunological outcomes that follow anthrax infection in humans.

## MATERIALS AND METHODS

### Test system.

NZW rabbits (Oryctolagus cuniculus, specific pathogen free) weighing 2.8 to 4.2 kg with implanted vascular access ports (VAPs) were procured from Covance Research Products, Inc. (Denver, PA).

### Study design.

The study was conducted in 2 phases, phase 1 and phase 2. The study was randomized, placebo-controlled, and parallel-group (50% male and 50% female, with exception of the control group in phase 2, which was 100% males). In phase 1, animals were randomized to treatment prior to spore challenge by weight and sex. Animals were then randomized to a challenge day and challenge order, such that there was approximately an equal number of animals from each group on each challenge day. In phase 2, phase 1 survivors and 12 additional naive control animals were randomized to a challenge day and challenge order. In both study phases, rabbits were exposed nose only to an aerosolized dose of B. anthracis spores (Ames strain), targeting 200 median lethal dose (LD_50_) (30) by real-time plethysmography. Phase 1 was open-label, but phase 2 was blinded. Prior to the start of phase 2, a transponder identification chip was implanted subcutaneously into the shoulder region of each rabbit that survived phase 1 to take the place of the ear tag identification. The transponder number differed from the phase 1 ear tag identification number, which was obscured.

### Treatment administration.

Levofloxacin (Levaquin oral solution; Ortho-McNeil, Raritan, NJ) was administered by gastric intubation once daily at 50 mg/kg of body weight/day for 3 days; this dose was shown to be efficacious and well tolerated when given at the first signs of infection to NZW rabbits (12). Obiltoxaximab (Elusys Therapeutics, Inc.) was administered with the first dose of levofloxacin or water as a single intravenous (i.v.) dose of 16 mg/kg. No treatment was administered in phase 2.

### Study conduct.

All studies were conducted at the biosafety level 3 facilities at the Battelle Biomedical Research Center, Columbus, OH, with the approval of Battelle's Institutional Animal Care and Use Committee.

### Pharmacodynamic measurements. (i) Blood collection.

Blood samples for pharmacodynamic measurements were taken from the medial auricular artery or the marginal ear vein. In phase 1, blood samples were collected from all animals at the 1 h ± 5 min post-first treatment time point to confirm the appropriate dosing. Starting on day 7 postchallenge (PC), samples were taken relative to the day of challenge. In phase 2, all PC blood samples were collected relative to the median challenge time ±1 h. Mean challenge times were calculated using the end times of the first and last animals challenged.

### (ii) Measurement of serum PA.

PA63 and/or PA83 sera were quantified using a validated (31, 32) sandwich enzyme-linked immunosorbent assay (ELISA) method, as previously described (12). Briefly, obiltoxaximab (Elusys Therapeutics, Inc.) was utilized as a capture reagent, followed by detection with goat anti-PA antiserum and horseradish peroxidase-conjugated anti-gamma-chain secondary antibody (IgG; Invitrogen; Carlsbad, CA). The assay did not detect PA20 nor PA bound to serum obiltoxaximab. The assay lower limit of quantitation (LLOQ) was 9.68 ng/ml, and the upper limit of quantitation (ULOQ) was 40,000 ng/ml.

### (iii) Measurement of bacterial burden (blood and tissue).

Bacterial burden was assessed by incubating cultures of blood samples for 16 to 24 h. Fresh blood samples were serially diluted 1:10, and each dilution was plated in triplicate. In addition, at the time of gross necropsy, small tissue sections of brain, spleen, liver, and bronchial lymph node were obtained, homogenized in phosphate-buffered saline (PBS), and streaked onto a solid agar plate. The plates were incubated at 37 ± 2°C for a minimum of 48 h, and B. anthracis colonies were enumerated to determine the bacterial tissue burden.

### (iv) Measurement of serum obiltoxaximab.

Serum obiltoxaximab was detected using a validated electrochemiluminescence (ECL) method utilizing the Meso Scale Discovery (MSD) platform. Free obiltoxaximab was captured by biotinylated recombinant PA63 (rPA63; List Biological Laboratories, Inc.) bound to an MSD streptavidin-coated plate, and ruthenylated goat anti-human antibody was used as the detection reagent. This immunoassay method selectively detects obiltoxaximab in rabbit serum due to the usage of an anti-human IgG detection reagent. Standard curve calibrators and quality control samples were prepared in rabbit serum. The generated signal was read on an MSD platform. The ECL units were collected and regressed using a 5-parameter logistic (5PL) (^1^/*y*^2^ weighting factor) regression.

### (v) Measurement of serum antibodies to PA and TNA.

Serum anti-PA IgG levels were quantified using a validated ECL method with the MSD platform. Biotinylated rPA63 (List Biological Laboratories, Inc.) bound to an MSD streptavidin-coated plate was used as a capture reagent, and ruthenylated protein A/G (Thermo Scientific, Waltham, MA) was used as the detection reagent. Standard curve calibrators and quality control samples were prepared in rabbit serum. The signal was read on an MSD platform, and the ECL units were regressed using a 5PL (^1^/*y*^2^ weighting factor) regression. The results were measured against obiltoxaximab as a standard. Both obiltoxaximab and endogenous rabbit anti-PA IgG can be detected in this assay. A cell-based toxin neutralization assay (TNA) was used to qualitatively assess the neutralizing antibodies to LT, as described previously (22). Briefly, a serial dilution of the test samples and controls was prepared in a separate plate, followed by the addition of LT and incubation to allow for LT neutralization by serum antibodies. The mixtures were transferred to the J774A.1 cell-containing plates and incubated to allow intoxication to proceed. Cell viability was determined colorimetrically using a tetrazolium salt, 3-[4, 5-dimethylthiazol-2-yl]-2,5-diphenyltetrazolium bromide (MTT) as the reporter or signal system, and the optical density (OD) values at 570 nm were read using a 690-nm reference wavelength. The TNA SAS program fitted the 7-point serial dilutions of the reference serum standard and test sample serum OD values to a four-parameter logistic-log (4PL) function, which was used to calculate the reportable values, 50% neutralization factor (NF_50_), and 50% effective dilution (ED_50_). The assay has 3 levels of acceptance criteria: (i) plate OD acceptance criteria based on an inspection of the OD values and with the % coefficient of variation (CV) for the OD values of each dilution of the reference standard (RS) required to be ≤20%, (ii) plate SAS acceptance criteria based on calculations from the SAS output, including the ED_50_ of the RS and quality controls (QC) and the parameterization of the RS and QC curves, (iii) test sample acceptance criteria ensuring that the results from the test sample were acceptable and only applied to individual results that were greater than the limit of detection (LOD) for the assay. If any of these acceptance criteria were not met for a plate, the entire plate failed and was repeated.

The TNA has two reportable values, ED_50_ and NF_50_. The ED_50_ is the reciprocal of the dilution of a serum sample that results in 50% neutralization of the LT. The neutralization capacity of each test serum in relation to that of the reference serum (50% neutralization factor [NF_50_]) is the quotient of the ED_50_ of the test sample (ED_50_TS) and the ED_50_ of the reference serum standard (ED_50_RS). The NF_50_ is calculated as follows: NF_50_ = ED_50_TS/ED_50_RS. The NF_50_ serves as a relative measure of toxin neutralization.

### Statistical considerations.

The primary endpoint was the survival rate, defined as the percentage of animals alive at the scheduled phase 2 termination. The survival rate in each of the groups was compared to the placebo (all challenged animals) using one-sided Boschloo exact test with a Berger-Boos correction of a gamma value of 0.001 (33). Animals did not receive treatment in phase 2, and their group assignments were predefined based on the primary challenge. Rechallenge was considered successful if the mortality rate in the rechallenge control population exceeded 90%. Survival rates were summarized for phase 1 based on the treatment administered and including only animals surviving to receive treatment. Analyses were conducted with R using the package “exact.” Exact 95% confidence intervals for differences in survival rates are based on the score statistic (Proc Freq of SAS version 9). Baseline characteristics (sex, weight, and anti-PA IgG levels) were summarized at phase 2 prechallenge using descriptive statistics. Descriptive statistics for serum PA, quantitative bacteremia, anti-PA IgG, and TNA levels were generated for all phase I and phase 2 time points. Anti-PA IgG and TNA levels were examined using analysis of variance (ANOVA) models fitted to each time point, and Tukey's multiple comparisons were used to test whether levels significantly differed between the groups.
